# Machine Learning Approach to Investigating Macrophage Polarization on Various Titanium Surface Characteristics

**DOI:** 10.34133/bmef.0100

**Published:** 2025-02-26

**Authors:** Changzhong Chen, Zhenhuan Xie, Songyu Yang, Haitong Wu, Zhisheng Bi, Qing Zhang, Yin Xiao

**Affiliations:** ^1^ School and Hospital of Stomatology, Guangdong Engineering Research Center of Oral Restoration and Reconstruction, Guangzhou Key Laboratory of Basic and Applied Research of Oral Regenerative Medicine, Guangzhou Medical University, Guangzhou 510182, China.; ^2^ School of Basic Medical Sciences, Guangzhou Medical University, Guangzhou 511436, China.; ^3^ Laboratory for Myology, Department of Human Movement Sciences, Faculty of Behavioural and Movement Sciences, Amsterdam Movement Sciences, Vrije Universiteit Amsterdam, 1081 BT Amsterdam, The Netherlands.; ^4^ School of Medicine and Dentistry & Institute for Biomedicine and Glycomics, Griffith University, Gold Coast, QLD 4222, Australia.

## Abstract

**Objective:** Current laboratory studies on the effect of biomaterial properties on immune reactions are incomplete and based on a single or a few combination features of the biomaterial design. This study utilizes intelligent prediction models to explore the key features of titanium implant materials in macrophage polarization. **Impact Statement:** This pilot study provided some insights into the great potential of machine learning in exploring bone immunomodulatory biomaterials. **Introduction:** Titanium materials are commonly utilized as bone replacement materials to treat missing teeth and bone defects. The immune response caused by implant materials after implantation in the body has a double-edged sword effect on osseointegration. Macrophage polarization has been extensively explored to understand early material-mediated immunomodulation. However, understanding of implant material surface properties and immunoregulations remains limited due to current experimental settings, which are based on trial-by-trial approaches. Artificial intelligence, with its capacity to analyze large datasets, can help explore complex material–cell interactions. **Methods:** In this study, the effect of titanium surface properties on macrophage polarization was analyzed using intelligent prediction models, including random forest, extreme gradient boosting, and multilayer perceptron. Additionally, data extracted from the newly published literature were further input into the trained models to validate their performance. **Results:** The analysis identified “cell seeding density”, “contact angle”, and “roughness” as the most important features regulating interleukin 10 and tumor necrosis factor α secretion. Additionally, the predicted interleukin 10 levels closely matched the experimental results from newly published literature, while the tumor necrosis factor α predictions exhibited consistent trends. **Conclusion:** The polarization response of macrophages seeded on titanium materials is influenced by multiple factors, and artificial intelligence can assist in extracting the key features of implant materials for immunoregulation.

## Introduction

Titanium is widely used as a clinical bone implant or bone replacement material due to its ideal mechanical properties and biocompatibility [1–3]. The immune response caused by titanium metal materials after implantation, especially the macrophage immune response, plays a double-edged sword effect on osseointegration and bone regeneration [4]. While an excessive immune reaction may result in peri-implantitis and treatment failure [5], an appropriately regulated immune response can promote successful osseointegration and tissue regeneration [6].

Macrophages exhibit 2 phenotypes depending on environmental cues: M1, which releases pro-inflammatory cytokines such as tumor necrosis factor α (TNF-α) that may impede bone healing, and M2, which promotes anti-inflammatory responses and tissue regeneration by secreting cytokines like interleukin 10 (IL-10) [7–9]. Given the significance of macrophages in mediating the immune response, modulating their polarization through the surface physicochemical properties of titanium has been a primary research focus. Several strategies, including surface modifications that adjust roughness, hydrophilicity, and pore size and modification of titanium metal surfaces through the introduction of copper, zinc, magnesium, and silver ions, have been explored to optimize immune regulation [10–16]. However, most studies focused on modifying one or a few titanium surface characteristics at a time, which is incomplete in unveiling the cumulative effects on the macrophage. The polarization response of macrophages is collectively influenced by various factors on the titanium surfaces, rather than by individual physicochemical properties [17,18].

Mathematical modeling has been employed to predict macrophage behavior and bone growth in response to titanium surfaces, yet these models cannot handle large-scale, complex datasets, limiting their predictive power [19,20]. To address this gap, advanced methods like machine learning (ML) can provide a more holistic and data-driven approach to studying the complexity of macrophage polarization and the immune response to titanium surfaces. Since its inception in the 1950s, artificial intelligence (AI) technology has evolved into a comprehensive field spanning computer science, logic, biology, psychology, philosophy, and more [21,22]. At its core, AI aims to extend human intelligence by training computers to simulate and learn humanlike behaviors [23]. ML, a branch of AI, has shown immense potential in analyzing large datasets and revealing complex patterns in fields like medicine and materials science [24]. In medicine, materials science, and their intersections, ML has become crucial for analyzing complex experimental data, predicting material properties, and optimizing experimental design. For instance, in medicine, ML aids in the early detection of osteoporosis by identifying abnormal bone density [25]. In materials science, it assists researchers in discovering novel polymers with high glass transition temperatures [26]. Moreover, at the intersection of materials science and medicine, ML is employed to predict the toxicity of metal oxide nanoparticles to immune cells, offering valuable insights for designing and applying safe nanoparticles [27]. The digitized ML approach surpasses traditional data acquisition, integration, and analysis techniques, enabling efficient extraction and exploration of correlations in complex datasets to assist researchers in prediction [28].

**Fig. 1.**
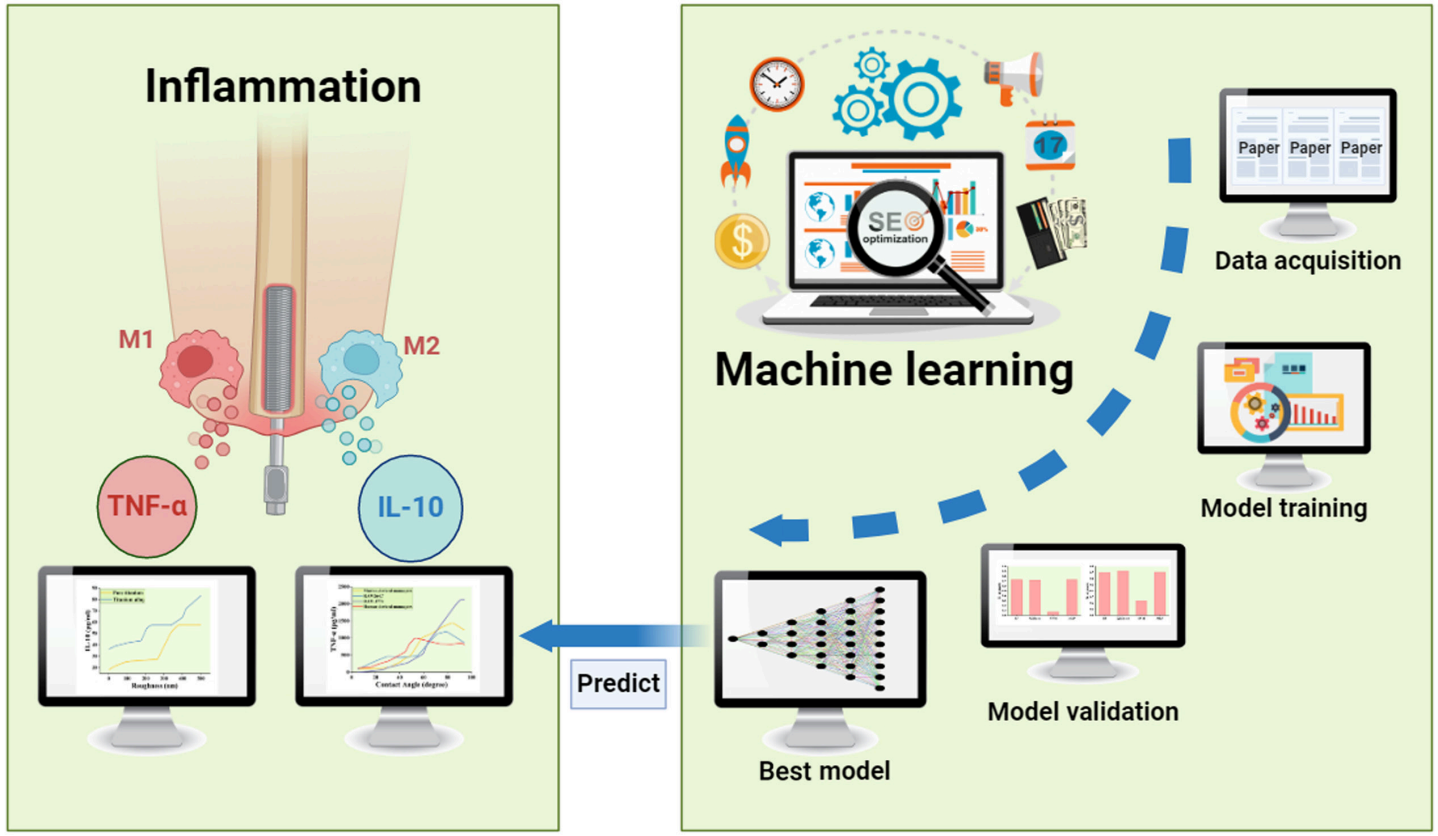
A diagram showing how machine learning aids in investigating the relationship between Ti implant surfaces and macrophage immune responses. TNF-α, tumor necrosis factor α; IL-10, interleukin 10.

By employing ML techniques to investigate the modulation of macrophage polarization, it is possible to conduct an in-depth analysis of titanium metal’s regulatory role (Fig. 1). IL-10 and TNF-α, as key anti-inflammatory and pro-inflammatory factors of macrophages, respectively, play essential roles in regulating immune responses [29–32]. This study explores the predictive and analytical capabilities of ML techniques in macrophage polarization by the surface physicochemical properties of titanium metal.

## Results

### Data collection

**Fig. 2.**
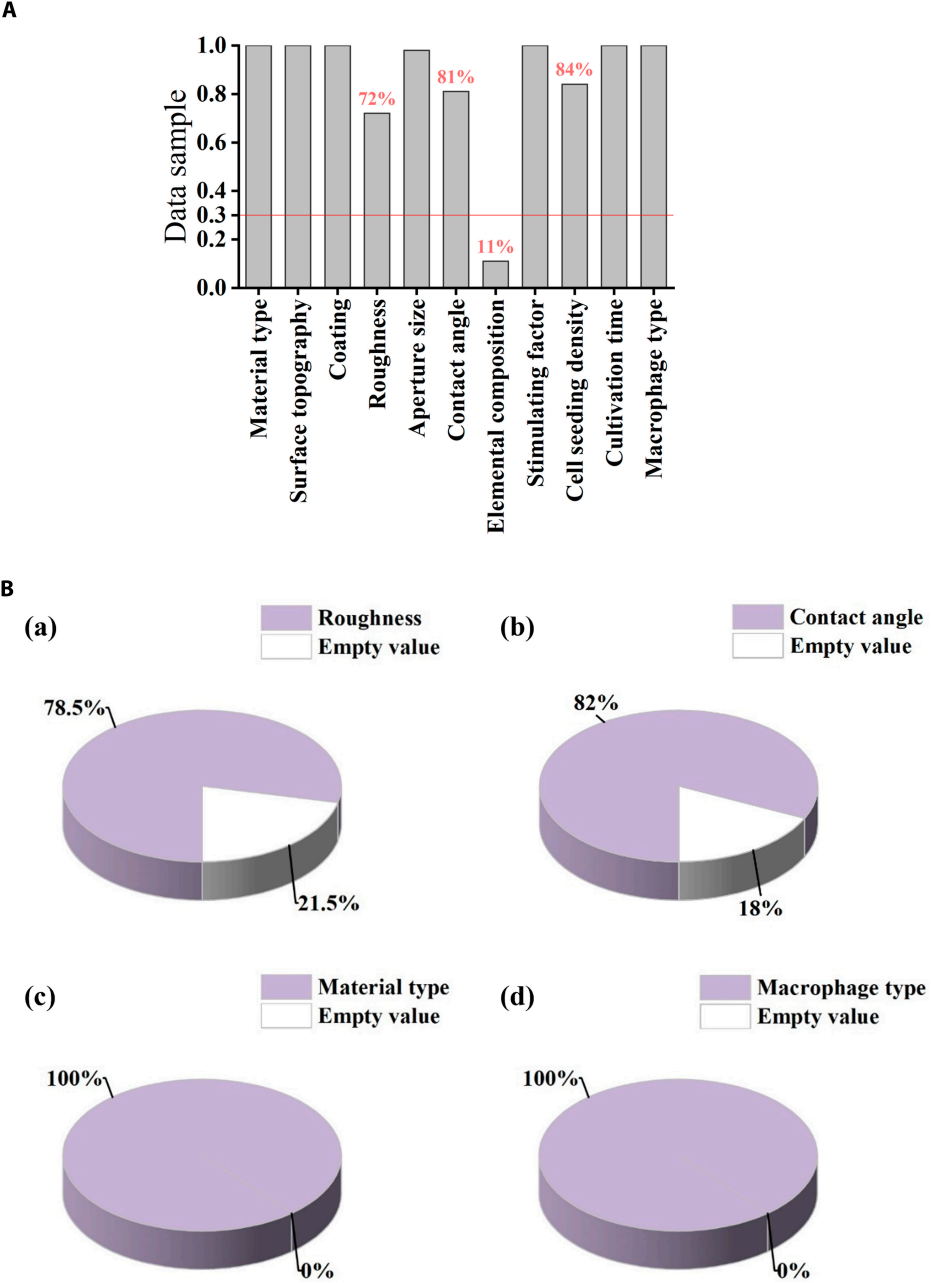
Diagrams of dataset distribution. (A) Bar chart illustrating the sample completeness rates across different features. (B) Pie charts showing the distribution of missing data for 4 features: (a) “roughness”, (b) “contact angle”, (c) “material type”. and (d) “macrophage type”.

To construct datasets suitable for effectively training ML models, this study curated and collected 128 academic papers related to the modulation of macrophage polarization by titanium metal materials. A rigorous screening process ultimately selected 35 papers that met the criteria for further analysis. Among these 35 papers, a total of 13 features related to the modulation of macrophage polarization by titanium metal materials were mentioned in common. According to the selection criteria for features, 3 features, namely, “elemental composition”, “temperature”, and “carbon dioxide concentration”, were excluded. As shown in Fig. 2, the data content of the “elemental composition” feature accounted for only 11% of the total data samples, which was significantly lower than the standard 30%. Additionally, the data samples of the “temperature” and “carbon dioxide concentration” features exhibited a single value, violating the standard “feature values cannot be unique”, and thus, these 2 features were excluded.

**Fig. 3.**
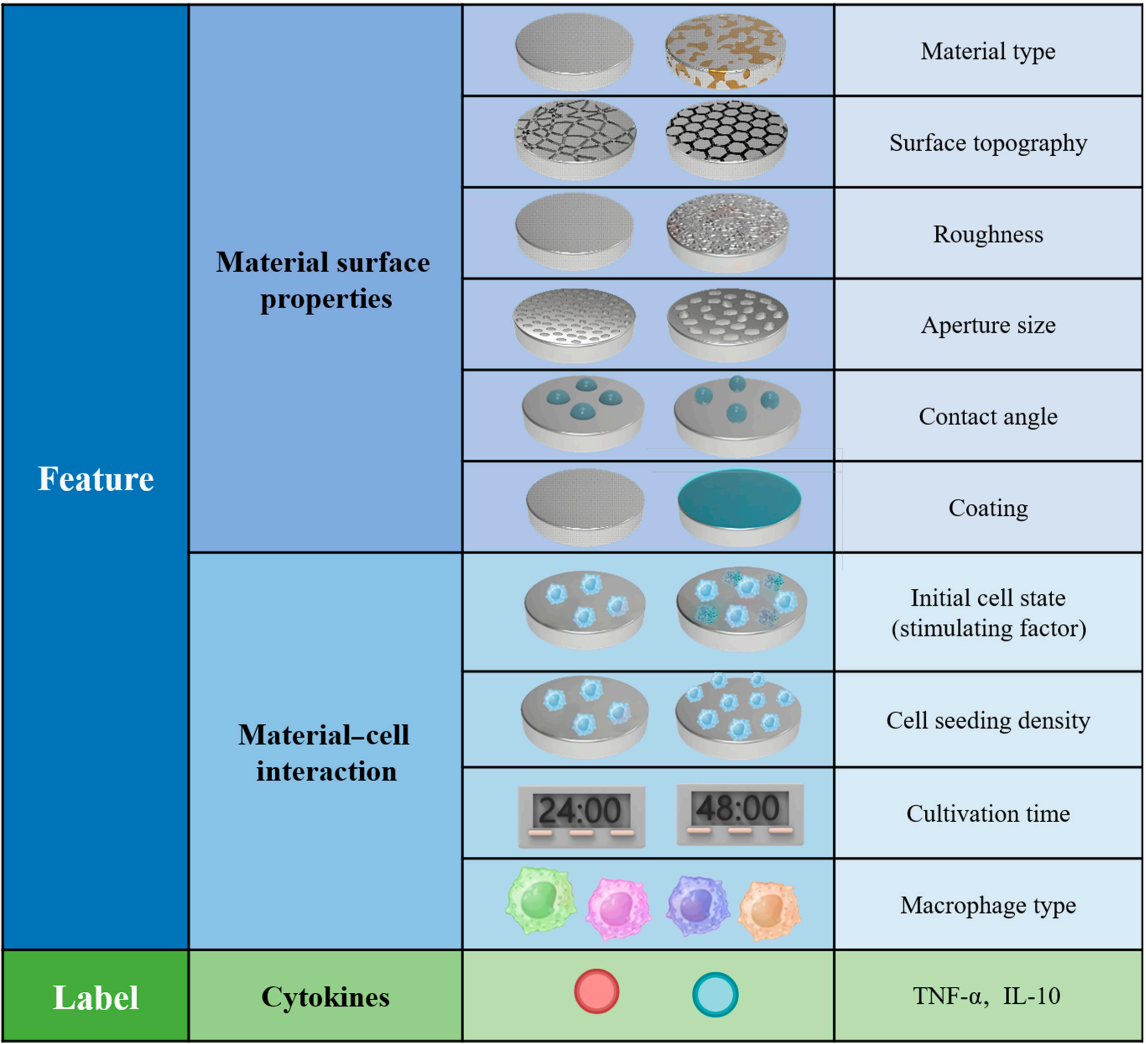
Relevant features and labels for experiments.

Following the data and feature selection, the IL-10 dataset and TNF-α dataset were successfully established, with the IL-10 dataset containing 167 data samples and 10 features and the TNF-α dataset containing 232 data samples and 10 features, as detailed in Fig. 3. Among these 10 features, “material type”, “surface topography”, “coating”, “roughness”, “aperture size”, and “contact angle” were related to the physicochemical properties of titanium metal surfaces, while “stimulating factor”, “cell seeding density”, “cultivation time”, and “macrophage type” involved the interaction between titanium materials and macrophages.

### Data preprocessing

To enhance the fitting performance of ML models, it is essential to improve the quality of data through preprocessing techniques. As depicted in Fig. 2B, the pattern of missing data varies across different features, providing valuable insights for effective data preprocessing. The missing rates of “roughness” and “contact angle” were 21.5% and 18%, respectively (Fig. 2B). The deletion rate of other characteristics was 0 (Fig. 2B). As a result, our entire dataset is characterized by less than 30% missing values, indicating that we have built a complete dataset that can be used for ML training and testing.

### Model training and selection

**Fig. 4.**
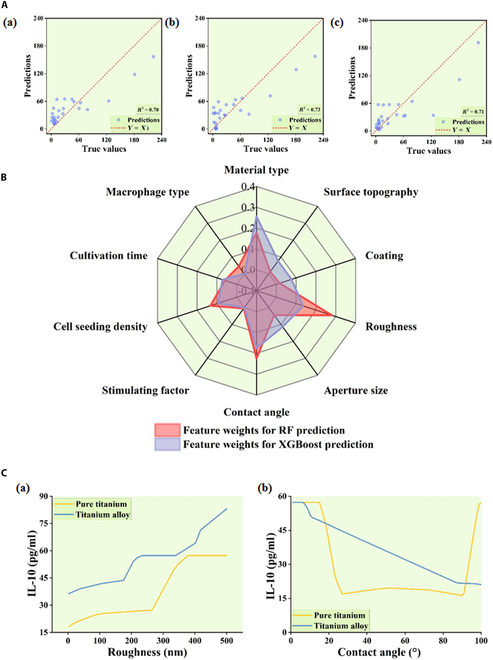
Machine learning model verification and prediction. (A) Scatter plot illustrating the comparison between actual and predicted values on the test set. The (a) RF, (b) XGBoost, and (c) MLP models predicted depict the predictions of the RF, XGBoost, and MLP models, respectively, compared to the true values on the test set of IL-10 dataset. (B) Radargram for predicting feature weights using the RF and XGBoost models. (C) Secretion levels of IL-10 predicted by the MLP model when macrophages are seeded on surfaces with different (a) roughnesses and different (b) contact angles on pure titanium and titanium alloy.

After training the models on the training set, it is necessary to evaluate the models using performance metrics to select the appropriate models for predictive applications. *R*-squared (*R*^2^) is a statistical metric utilized to quantify the proportion of variability in the dependent variable that is explained by the independent variables in regression models. Mean absolute percentage error (MAPE), on the other hand, reflects the accuracy of model predictions and is applicable for evaluating regression model performance, with its value range being (0,+∞). By comprehensively considering both *R*^2^ and MAPE, the performance of the models can be evaluated from 2 dimensions: model fit and prediction accuracy. The performance differences among the 4 ML models on the IL-10 dataset’s test set are illustrated in the Table. Notably, the random forest (RF) (*R*^2^ = 0.70, MAPE = 2.82), extreme gradient boosting (XGBoost) (*R*^2^ = 0.73, MAPE = 3.60), and multilayer perceptron (MLP) (*R*^2^ = 0.71, MAPE = 1.69) models outperform the support vector machine (SVM) model (*R*^2^ = 0.24, MAPE = 3.64), with *R*^2^ values surpassing 0.7 for these 3 models. In this study, *R*^2^ values exceeding 0.7 indicate that the features considered in the models can explain over 70% of the variability in cytokine secretion response. Higher *R*^2^ values signify better fitting of the model to the observed data. Therefore, the selected ML models, including RF, XGBoost, and MLP, demonstrated significant capabilities in capturing and interpreting latent patterns relating the selected features to cytokine secretion responses. Furthermore, Fig. 4 illustrates the comparison between the predicted results and actual values of the RF, XGBoost, and MLP models on the test set of the IL-10 dataset. In the 3 scatter plots of Fig. 4A, the majority of data points are distributed around the diagonal, with only a few data points deviating noticeably from the diagonal line. Hence, from the performance on the test set, the RF, XGBoost, and MLP models demonstrate predictive capabilities simultaneously.

**Table.** *R*-squared (*R*^2^) and MAPE values for 4 machine learning models on the IL-10 dataset test set

ML models	*R* ^2^	MAPE
RF	0.70	2.82
XGBoost	0.73	3.60
SVM	0.24	3.64
MLP	0.71	1.69

MAPE, mean absolute percentage error; ML, machine learning; RF, random forest; XGBoost, extreme gradient boosting; SVM, support vector machine; MLP, multilayer perceptron

### Model prediction

To explore the key features regulating macrophage secretion of IL-10, the RF and XGBoost models, which exhibited high fitting performance on the test set, can be utilized for prediction. RF and XGBoost models are ensemble algorithms based on tree models, possessing high interpretability. As depicted in Fig. 4B, both the RF and XGBoost models identified “cell seeding density”, “contact angle”, “material type”, and “roughness” as significant features influencing IL-10 protein secretion.

Due to the excellent fitting performance of the MLP model on the test set and the lowest MAPE value, the MLP model can be further employed to investigate the specific impact of key features on IL-10 secretion levels. As shown in Fig. 4C (a), the trend of IL-10 secretion levels predicted by the MLP model, with the change in surface roughness, was similar for macrophages seeded on pure titanium and titanium alloy, both exhibiting an increase in IL-10 secretion levels with increasing roughness. Simultaneously, macrophages seeded on titanium alloy generally exhibited higher IL-10 secretion levels compared to those seeded on pure titanium. Additionally, as illustrated in Fig. 4C (b), according to the MLP model’s prediction, significant differences in IL-10 secretion levels were observed for macrophages seeded on pure titanium and titanium alloy under different values of “contact angle”. When the water contact angle ranged from 40° to 80°, the IL-10 secretion levels of macrophages seeded on titanium alloy decreased with an increase in the water contact angle, while the IL-10 secretion levels of macrophages seeded on pure titanium remained relatively stable.

### Experimental verification

**Fig. 5.**
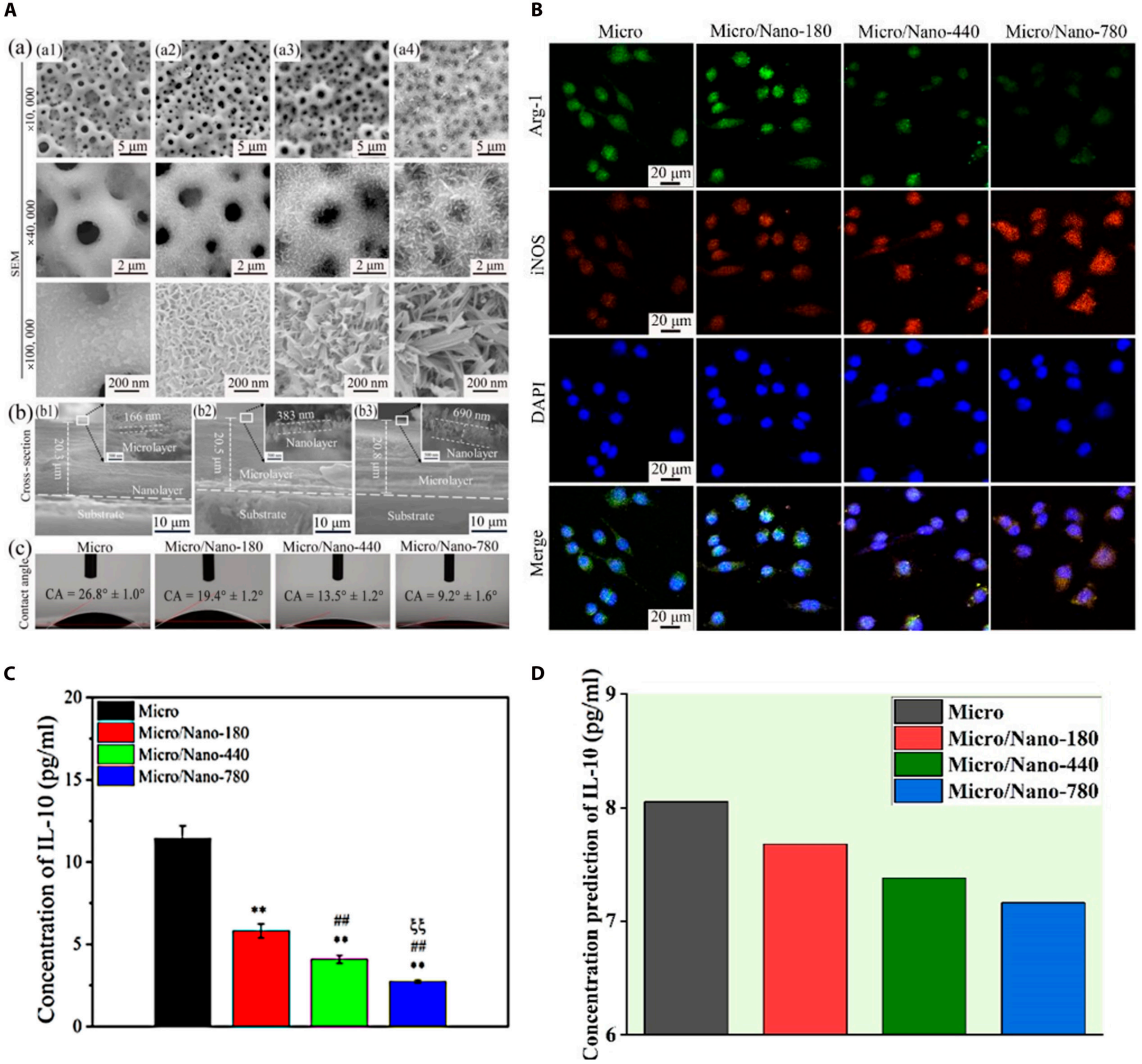
Application of machine learning predictive models. (A) As reported in the literature [33], scanning electron microscopy (SEM) images showing the (a) surface and (b) cross-sectional morphology of (a1) micro-arc-oxidation (MAO)-treated Ti without and with hydrothermal treatment (HT) for (a2 and b1) 8, (a3 and b2) 36, and (a4 and b3) 72 h; (c) the contact angles (CAs) of various surfaces. Source: Reprinted with permission from Ref. [39]. Copyright 2021, with permission from Elsevier. (B) As reported in the literature [33], immunofluorescent staining images showing the expressions of arginase 1 (Arg-1) (green, M2 marker) and inducible nitric oxide synthase (iNOS) (red, M1 marker) as well as the presence of cell nuclei (blue) in macrophages grown on various surfaces. Source: Reprinted with permission from Ref. [39]. Copyright 2021, with permission from Elsevier. (C) Bar chart illustrating the true secretion levels of IL-10 by macrophages as reported in the literature [33]. **Compared with the Micro group, *P* < 0.01; ^##^compared with the Micro/Nano-180 group, *P* < 0.01; ^ξξ^compared with the Micro/Nano-440 group, *P* < 0.01. Source: Reprinted with permission from Ref. [39]. Copyright 2021, with permission from Elsevier. (D) Bar chart illustrating the predicted secretion levels of IL-10 by macrophages using the MLP model. DAPI, 4′,6-diamidino-2-phenylindole.

To further validate the practicality of the MLP model, an important step involves testing its predictive performance with new experimental data. Through literature research, this study selected a literature piece showcasing comprehensive feature data that are highly relevant to the research topic [33]. In this experiment, micro–nanostructured surfaces were fabricated on titanium substrates using the micro-arc oxidation technique, denoted as “Micro”. Subsequently, using the hydrothermal technique, platelike nanostructures with average lengths of 180, 440, and 780 nm were prepared on the “Micro” surface, designated as “Micro”, “Micro/Nano-180”, “Micro/Nano-440”, and “Micro/Nano-780”, respectively. Figure 5A illustrates the surface morphology and physicochemical properties of these 4 different surfaces, where the hydrophilicity of the surfaces increases with the length of the nanoplates. Figure 5B illustrates the expression of arginase 1 and inducible nitric oxide synthase by macrophages on these 4 different surfaces using immunofluorescence staining, indicating that these distinct surface structures can modulate macrophage polarization response to varying degrees. Figure 5C presents the actual secretion levels of IL-10 by macrophages seeded on the 4 different surfaces in the literature experiments, while Fig. 5D shows the predicted IL-10 secretion levels of macrophages on the 4 different surfaces using the MLP model constructed in this study. The results demonstrate a high consistency between the predicted results and the experimental results from the literature, indicating the high reliability of the MLP model in predicting IL-10 secretion levels.

To validate the universality of ML in exploring both the physicochemical properties of titanium metals and the mutual modulation of titanium metals and macrophages on macrophage polarization, this study established a TNF-α dataset. RF, XGBoost, SVM, and MLP models were constructed using this dataset to predict TNF-α secretion levels, and excellent models were selected based on evaluation metrics such as *R*^2^ and MAPE for predictive applications.

**Fig. 6.**
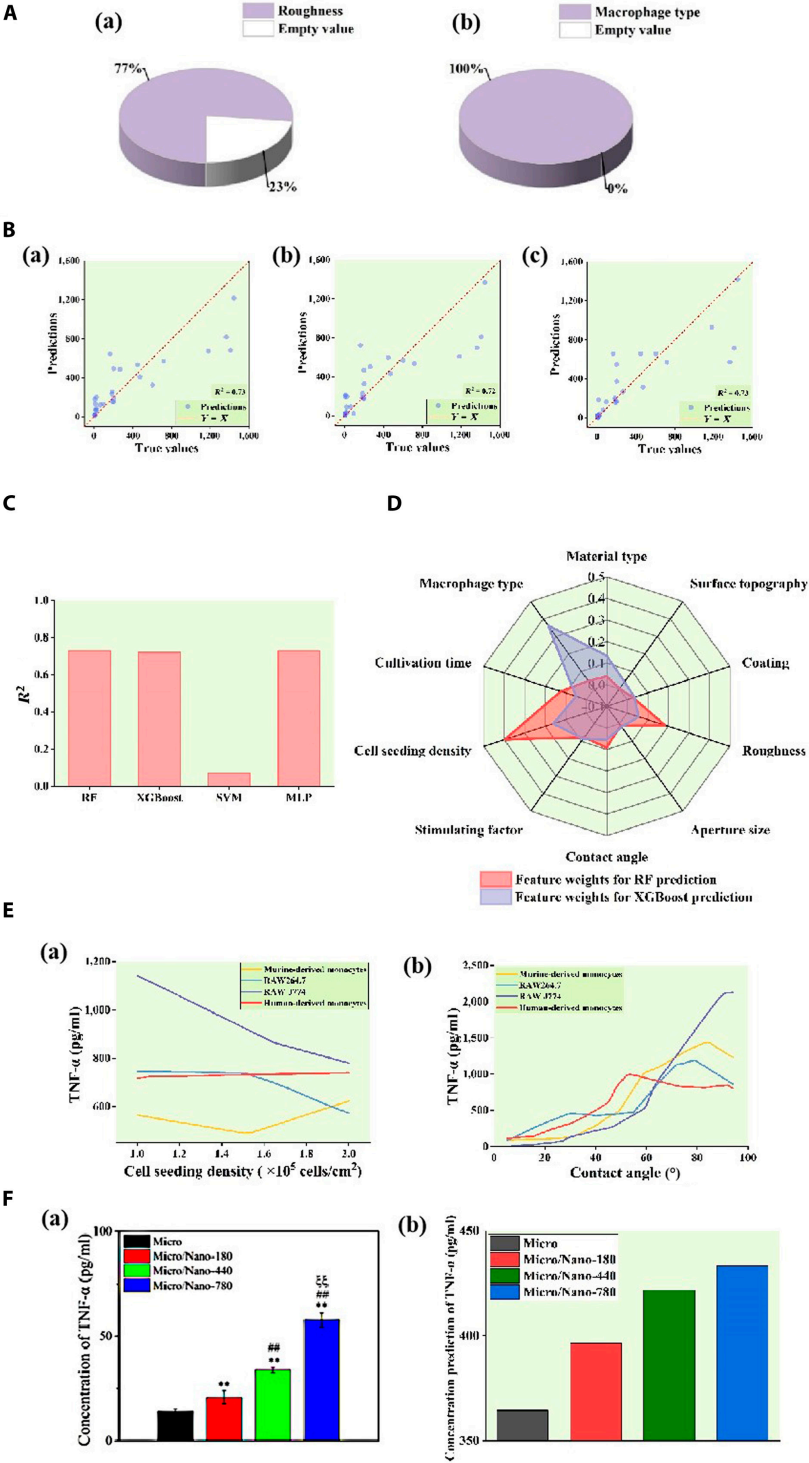
Validation and application of the machine learning prediction model for TNF-α secretion. Construction of a machine learning model for predicting the level of TNF-α secretion in macrophages regulated by titanium materials using machine learning techniques. (A) Description of missing data for the features (a) “roughness” and (b) “macrophage type” in the TNF-α dataset. (B) Test set samples were predicted using the (a) RF, (b) XGBoost, and (c) MLP models on the TNF-α dataset and compared to the true values. (C) *R*^2^ comparison bar charts of the RF, XGBoost, SVM and MLP models on the test set of the TNF-α dataset. (D) Radargram for predicting feature weights using the RF and XGBoost models on the TNF-α dataset. (E) The TNF-α secretion levels of 4 macrophage species are demonstrated. (a) Levels of TNF-α secretion by macrophages when inoculated with titanium materials at different seeding densities. (b) Levels of TNF-α secretion in macrophages inoculated onto different hydrophilic titanium surfaces. (F) (a) Bar chart illustrating the true secretion levels of TNF-α by macrophages as reported in the literature [33]. **Compared with the Micro Group, *P* < 0.01; ^##^compared with the Micro/Nano-180 Group, *P* < 0.01; ^ξξ^compared with the Micro/Nano-440 group, *P* < 0.01. Source: Reprinted with permission from Ref. [39]. Copyright 2021, with permission from Elsevier. (b) Bar chart illustrating the predicted secretion levels of TNF-α by macrophages using the MLP model.

The initial step involved visualizing the extent of missing data in the TNF-α dataset, with Fig. 6A illustrating the absence of data for the features “roughness” and “macrophage type”. Subsequently, the data underwent preprocessing, including imputation of missing values using methods such as mode and *k*-nearest neighbor (kNN) algorithm, one-hot encoding for discrete features, and normalization for numerical features. The dataset was then divided into a training set (80% of the total data) and a test set (20% of the total dataset).

The preprocessed dataset was then utilized to train ML models. The comparison between the predicted values and the actual values on the test set for the RF, XGBoost, and MLP models is shown in Fig. 6B, where the data points are scattered around the diagonal, indicating high reliability for the 3 models. Therefore, the RF and XGBoost models, which are interpretable and perform well on the test set, were chosen in this study to predict the weights of factors affecting TNF-α protein secretion.

As depicted in Fig. 6C, the RF (*R*^2^ = 0.73, MAPE = 1.85), XGBoost (*R*^2^ = 0.72, MAPE = 1.96), and MLP models (*R*^2^ = 0.73, MAPE = 1.58) outperformed the SVM model (*R*^2^ = 0.07, MAPE = 3.65) on the test set of the TNF-α dataset. As shown in Fig. 6D, both the RF and XGBoost models identified “cell seeding density”, “contact angle”, and “roughness” as influential features for TNF-α protein secretion while considering “aperture size” to have a minor impact. Due to the high *R*^2^ and low MAPE of the MLP model, it was employed to explore the influence of key features on TNF-α secretion levels by macrophages. For example, as shown in Fig. 6E (a), the trends of TNF-α secretion levels by different types of macrophages varied with cell seeding density, with human-derived monocytes generally exhibiting higher TNF-α secretion levels than murine-derived monocytes. Additionally, as shown in Fig. 6E (b), macrophage TNF-α secretion levels were also influenced by the contact angle, with TNF-α levels increasing as the contact angle increased. Notably, RAW J774 cells exhibited a sharper increase in TNF-α levels compared to other macrophage types, particularly in the contact angle range of 60° to 90°. This further indicates that multiple factors regulate TNF-α secretion levels by macrophages.

Additionally, this study verified the utility of the MLP model using the literature described in the “Experimental verification” in Materials and Methods. Using the MLP model to predict the TNF-α secretion levels of macrophages seeded on 4 different surfaces mentioned in the literature, the predicted values were compared with the reported TNF-α secretion levels. The comparison results in Fig. 6F indicate that while significant discrepancies exist between the predicted and observed values, the predicted trend aligns with the observed values. Specifically, the predicted TNF-α secretion levels increase sequentially in the 4 experimental groups “Micro”, “Micro/Nano-180”, “Micro/Nano-440”, and “Micro/Nano-780”. This suggests that despite the significant differences, the MLP model has the potential to predict TNF-α secretion levels due to its ability to capture the underlying trend.

In summary, applying ML techniques to predict TNF-α secretion levels further demonstrates the high potential of ML techniques in the field of biomaterials.

## Discussion

This study explored ML approaches to extracting titanium metal characteristics that affect the properties of macrophage polarization and cytokine production, utilizing IL-10 and TNF-α as representative anti-inflammatory and pro-inflammatory agents, respectively.

In both the IL-10 and TNF-α datasets, the RF and XGBoost models consistently indicated that features such as “cell seeding density”, “contact angle”, and “roughness” significantly influence cytokine secretion. This finding aligns with numerous research outcomes, further emphasizing the importance of titanium metal surface hydrophilicity and its synergistic interaction with surface roughness [34,35]. It underscores the utility of RF and XGBoost models in our study’s predictive tasks. Moreover, the predictive results of RF and XGBoost models also highlight the crucial role of the “cell seeding density” feature in regulating macrophage secretion on titanium metal materials, reminding researchers to pay attention to experimental operational details. In both the IL-10 and TNF-α datasets, the MLP model’s predictions regarding the modulation of macrophage polarization responses by features such as “roughness”, “contact angle”, and “cell seeding density” are consistent with the findings of many researchers, such as the superior anti-inflammatory properties of titanium alloys compared to those of pure titanium, the better performance of highly hydrophilic surfaces in alleviating inflammatory responses, and the differences in experimental results obtained from different types of macrophages [34,36–38]. This further confirms the accuracy and practicality of the MLP model in prediction.

This study utilized the MLP model to predict the secretion levels of IL-10 and TNF-α on these modified surfaces. The results indicated a high level of agreement between the IL-10 secretion levels predicted by the MLP model and the experimental results in the literature. In contrast, TNF-α secretion levels showed similar trends but with significant numerical discrepancies. These discrepancies can be attributed to 4 main factors. Firstly, the limited data used to train the ML model resulted in features such as “elemental composition” not being included in the model due to data scarcity, despite their potential importance in modulating macrophage secretion levels. Secondly, variability in the experimental data, including experimental errors from the cited literature or potential errors made by experimenters in recording data, could have affected the model’s accuracy. Thirdly, range errors occurred during data input, as the model only accepted specific values without accounting for the reported variability. Fourthly, the data established through the literature search were unevenly distributed. For instance, few samples had TNF-α secretion levels above 400 pg/ml, limiting the model’s ability to capture responses under special conditions. Despite these biases, the predictive value of the MLP model in biological experiments was still validated.

Studies on the effects of different sizes of TiO_2_ nanotubes on macrophage polarization responses have yielded diverse conclusions in recent years. Notably, ML models trained in this study contribute to elucidating these seemingly contradictory research findings. For example, Lu et al. [16] suggested that 80-nm TiO_2_ nanotubes could better reduce in vitro verification, while Wang et al. [10] proposed that 80- to 100-nm TiO_2_ nanotubes could induce macrophages into the M1 phenotype, and 30-nm TiO_2_ nanotubes tended to induce M2 phenotype. These contradictory outcomes underscore the complexity of the interactions between titanium surface properties and macrophage behavior. This study utilized ML technology to explore the possible reasons behind these differences in depth. According to the predictions of RF and XGBoost models, the “cell seeding density” feature significantly influences macrophage polarization responses. At the same time, the “macrophage type” feature also has a certain impact on macrophage polarization. The differences in experimental operational details, such as the seeding density of macrophages and the selection of different types of macrophages by researchers on both sides, may be the reasons for the different results. These findings elucidate potential factors contributing to the observed disparities in previous studies. This study not only addresses the existing contradictions in the literature but also highlights the critical role of experimental details, such as macrophage seeding density and the selection of macrophage types, in shaping research outcomes in the field of implant materials.

The efficient data acquisition method, through extensive literature searches and in-depth reading, provided a wealth of experimental data to train ML models. However, this approach has limitations. Firstly, during the literature review process, certain features, such as “elemental composition”, were mentioned infrequently in the relevant studies in this field. Therefore, they were not included in the final feature selection. In this study, we reviewed the literature to summarize the relevant features reported in this field and statistically analyzed their occurrence frequency to determine which features to include. However, while this feature may influence macrophage polarization, the ML model could still capture its effects indirectly through other related features. Consequently, although some features were not explicitly used, their impact could be represented through correlations with other included features. Secondly, some literature is needed to provide complete feature data, leading to occurrences of missing data. For instance, during the thorough reading of a paper by Xu et al. [39], it was found that the feature “roughness” did not provide detailed data. Among the methods for handling missing values, simple approaches like replacing missing values with zeros, the mean, or the median are commonly used. These methods are straightforward, easy to implement, and computationally inexpensive, making them suitable when the missing data are minimal and the data distribution is uniform. In contrast, the kNN algorithm is highly effective for imputing missing values in cases with complex data patterns, especially when the missing data show some correlations. Even though this study employed mode and kNN algorithms to impute missing values, these filled data may only partially reflect the actual situation, which could affect the fitting of ML models trained on this dataset. Thirdly, when acquiring data from previous research literature, issues such as experimental personnel errors, inaccurate data recording, or errors in the experiments themselves may arise. For example, in a study by Zhang et al. [40], the data for the feature “contact angle” exhibited a range of errors, with the “contact angle” data for the different groups Ti, Ti-A, Ti-D, and Ti-AA recorded as 96.80° ± 2.08°, 14.37° ± 2.00°, 50.11° ± 2.40°, and 26.22° ± 1.50°, respectively. Therefore, there is a need to improve the methods used to collect data in order to increase the quality and reliability of the datasets used for ML model training and validation.

Through the predictive analysis of RF and XGBoost models, this study revealed the significant impact of titanium surface roughness, hydrophilicity, and the operational detail of the seed density on the in vitro polarization response of macrophages. This suggests that researchers should enhance their focus on these physicochemical properties and experimental procedures in future studies. Additionally, the MLP model predicted the effects of variations in key features on the secretion levels of cytokines by macrophages, aiding in elucidating the complex relationship between these key features and cytokine secretion levels. These findings provide valuable insights for deepening researchers’ understanding of the intricate interactions between titanium surface properties and immune responses.

This study aimed to investigate the synergistic regulation of titanium surface properties and titanium–macrophage interactions on macrophage polarization responses using ML technology, focusing on the secretion of the cytokines IL-10 and TNF-α. Through the application of RF and XGBoost algorithms, significant influences of the features “cell seeding density”, “contact angle”, and “roughness” on macrophage polarization responses were identified. Particularly noteworthy was the prominent effect of the feature “cell seeding density”, highlighting the importance of the physicochemical properties of titanium and its interaction with macrophages, thus emphasizing the need for increased attention to experimental operational details. Using the MLP model, variations in the key features “cell seeding density”, “contact angle”, and “roughness” on IL-10 and TNF-α secretion levels were explored, providing deeper insights into the complex relationships between titanium surface physicochemical properties, titanium–macrophage interactions, and macrophage polarization responses. For instance, it was observed that when macrophages were seeded on highly hydrophilic pure titanium and titanium alloy surfaces, higher levels of IL-10 secretion were observed. In contrast, differences in IL-10 secretion levels were noted when macrophages were seeded on pure titanium and titanium alloy surfaces with water contact angles between 40° and 80°. ML techniques were employed to elucidate the apparent contradictions among recent research findings in the field, demonstrating the wide-ranging potential applications of ML in the interdisciplinary field of biomaterials.

Future work in this area can further explore the applications of ML in the field of biomaterials from several aspects. Firstly, to obtain more diverse and high-quality data for building highly fitting ML models and comprehensively analyzing the relationships between features and labels, future studies should adopt high-throughput experimental methods for data collection. Secondly, regarding model selection, ML models that are robust with small datasets, such as RF, XGBoost, MLP, and SVM, were utilized in this study due to the relatively small sample size. Given the stringent requirements for interpretability in medical research, these models can ensure reliable and understandable predictions. Integrating advanced ML techniques and algorithms in future research could significantly improve the models’ ability to capture the complex and nonlinear relationships between features and labels, thereby enhancing predictive performance. Techniques such as deep learning, ensemble methods, and transfer learning will be particularly beneficial. Lastly, future research should focus on a deeper mechanistic understanding, exploring how different physicochemical properties of implanted materials and their cell interactions influence cytokine secretion through cellular signaling pathways.

In conclusion, this study explored the potential application of ML technology in the field of biomaterials, deepening our understanding of the complex interactions between titanium surface properties and immune responses.

## Materials and Methods

**Fig. 7.**
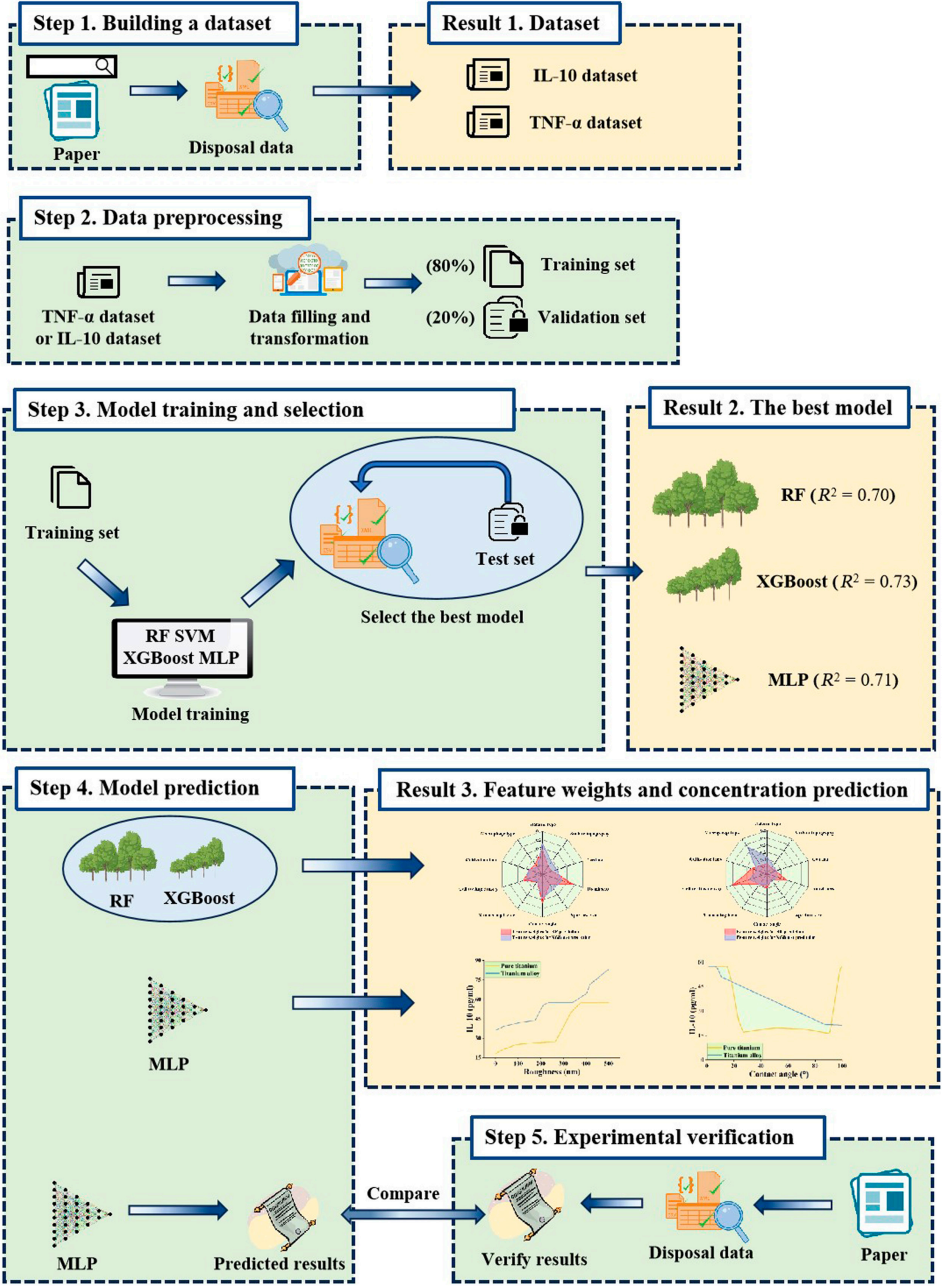
Overview of the study workflow and key findings. In step 1, datasets for IL-10 and TNF-α were created by systematically searching the relevant literature in public databases and selecting appropriate data. Result 1 highlights the obtained datasets. In step 2, data preprocessing was performed, including data filling and transformation, followed by partitioning the datasets into 80% for training and 20% for validation. In step 3, RF, XGBoost, SVM, and MLP models were trained on the training dataset using 10-fold cross-validation, followed by performance evaluation on the test set to identify the best performing model. Result 2 shows that XGBoost achieved the highest *R*^2^ value (0.73), followed by MLP (0.71) and RF (0.70). In step 4, the chosen models were used to predict IL-10 secretion levels, with feature weights and concentration prediction results shown in result 3. In step 5, experimental verification was performed by comparing the predicted results with new experimental data obtained from the literature, ensuring that model predictions were consistent with experimental outcomes.

The study workflow is presented in Fig. 7.

### Building datasets

The data incorporated in this study primarily originated from previous research papers and experimental findings, meticulously curated through an in-depth literature review to ensure the inclusion of accurate information. Data from figures were extracted using the software GetData Graph Digitizer 2.24 (http://getdata1.software.informer.com). Initially, a comprehensive search of literature from 2002 to 2021 was conducted on Google Scholar, Web of Science, and PubMed using keywords such as “titanium surface modification”, “nanomodification”, “macrophage polarization”, “inflammation”, “titanium dioxide”, and “immune response”. Subsequently, the literature was screened based on 4 criteria: (a) Experiments selected from the literature must be conducted in vitro. (b) The selected literature must exclusively explore the modulation of macrophage polarization by titanium surface physicochemical properties, prohibiting any other types of experiments, such as studies involving the addition of drugs to titanium for regulating macrophage polarization. (c) The experiments selected from the literature are not co-culture experiments, and the substrate material for seeding macrophages must be titanium or titanium alloy. (d) Studies on macrophage polarization reactions in the selected literature must include enzyme-linked immunosorbent assay experiments. Finally, feature data information was extracted from the screened literature to establish datasets with target labels representing the secretion levels of IL-10 and TNF-α by macrophages, named as the IL-10 dataset and TNF-α dataset, respectively. The criteria for feature selection were as follows: (a) The data sample size must exceed 30% of the total sample size. (b) Feature values cannot be unique.

### Data preprocessing

When extracting data from the literature, numerical features were recorded directly to ensure accuracy. For instance, the “contact angle” feature, representing water contact angle values, was documented as presented in the papers. In cases where different papers reported values in varying units, these were converted to a common unit to ensure consistency before being recorded. All values were carefully documented to reflect the original measurements accurately. For discrete features, such as “material type”, which could take values like “pure titanium” or “titanium alloy”, standard labels were assigned for consistency. Specifically, “A” was used for pure titanium and “B” for titanium alloy. This labeling method was employed to facilitate the transformation of categorical data into formats suitable for ML models. To ensure effective training of ML models, data preprocessing was conducted prior to model training in this study. Firstly, visualization of missing values for features was performed to understand the extent of missing data, providing a basis for subsequent data analysis. Next, the dataset was split into 2 groups based on the label values, specifically one dataset with TNF-α secretion levels as the label and the other with IL-10 secretion levels as the label. Missing values were handled according to feature type. Within each group, missing values of discrete features were imputed using the mode, while missing values of numerical features were filled using the kNN regression method, with *k* = 5. The algorithm identifies the 5 closest data points based on the feature space and assigns the missing value by averaging the nearest neighbors’ values. Subsequently, one-hot encoding was employed to preprocess discrete features, thereby converting them into numerical representations suitable for ML algorithm training. For numerical features, standardization was applied to preprocess the data, ensuring uniform scaling across features. Finally, the dataset was randomly split, allocating 80% for training the models and adjusting model hyperparameters, and 20% for testing the model’s performance.

### Model training and selection

To predict the secretion levels of IL-10, a numerical data type, 4 suitable ML models were selected: RF, XGBoost, SVM, and MLP models. Model training was conducted in Python using the scikit-learn package within the Jupyter environment. The specific experimental steps were as follows: (a) The data of the training set were randomly divided into 10 subsets for subsequent 10-fold cross-validation. (b) Hyperparameter tuning for the 4 models was performed using a combination of 10-fold cross-validation and grid search strategy. Grid search is a widely used hyperparameter optimization method aimed at finding the best combination of hyperparameters to enhance model performance and generalization ability.

Evaluation of the trained models in this study involved 2 main aspects: assessing the performance differences of the 4 models on the dataset and evaluating their practical application performance. For performance difference evaluation, the Wilcoxon signed-rank test was conducted for statistical validation, while assessment of practical application performance was carried out by testing the effectiveness on an independent test set to identify the best-performing model.

### Model prediction

To further explore the role of titanium surface physicochemical properties and the interaction between titanium and macrophages in regulating the secretion of IL-10 by macrophages, this study employed the models with excellent performance on the test set to conduct predictions: ranking the weights of factors influencing the protein secretion of IL-10 in macrophages and investigating the influence of features on IL-10 protein secretion under different conditions.

### Experimental verification

In this section, the validation of the selected optimal model’s practicality in real-world applications and the exploration of ML techniques’ generalizability in this domain are addressed. The experimental verification entails 2 main aspects: firstly, assessing the model’s performance in predicting IL-10 secretion levels and, secondly, constructing predictive models using ML techniques to forecast TNF-α secretion levels.

To validate the practicality of the selected optimal model in real-world applications, this study conducted searches on Google Scholar, Web of Science, and PubMed databases to retrieve relevant literature on the modulation of macrophage polarization by titanium surface physicochemical properties. The search employed keywords and filtering criteria consistent with those outlined in the “Building datasets” section, albeit limited to articles and experiments published after 2021. After an in-depth review of these articles, those with the most comprehensive data and closely related to the experiments of this study were selected. Subsequently, the feature data extracted from the identified literature were input into the optimal model to predict the secretion level of IL-10. Finally, the predicted results were compared and analyzed against the actual values measured in the literature to evaluate the performance of the selected model in real-world application scenarios.

Additionally, to explore the applicability of ML in this domain, similar research methodologies were employed in this study to investigate the influence of titanium surface physicochemical properties and macrophage interactions on the secretion of macrophage TNF-α. Specifically, through literature collection and screening, data were extracted and datasets were constructed to train ML models to predict the secretion of the pro-inflammatory cytokine TNF-α. The operational steps were as follows: (a) Following the experimental methods outlined in the “Data preprocessing” section in Materials and Methods, data preprocessing was conducted on the TNF-α dataset. (b) Following the experimental methods outlined in the “Model training and selection” in Materials and Methods, ML models were constructed, and excellent models were evaluated and selected using a test dataset. (c) Following the experimental methods outlined in the “Model prediction” in Materials and Methods, the constructed ML models were applied to predict the importance of features and explore the specific effects of key features on TNF-α secretion levels. (d) The selected optimal models were utilized to predict TNF-α secretion levels in the experimental literature selected in this section, and the predicted values were compared with the actual TNF-α secretion levels reported in the literature to further validate the practicality of the models.

## Data Availability

All data needed to evaluate the conclusion of this study are available on request.
